# EEG Spectral Analysis in Chronic Pain During Rest and Cognitive Reasoning

**DOI:** 10.3390/s25196230

**Published:** 2025-10-08

**Authors:** Diana Chertic, Victor Dăbală, Livia Livinț-Popa, Maria Balea, Nicu Cătălin Drăghici, Ștefan Strilciuc, Răzvan Cherecheș, Vitalie Văcăraș, Dafin F. Mureșanu

**Affiliations:** 1 Department of Neuroscience, “Iuliu Hațieganu” University of Medicine and Pharmacy, 400083 Cluj-Napoca, Romania; diana.chertic@brainscience.ro (D.C.); victor.dabala@brainscience.ro (V.D.); stefan.strilciuc@brainscience.ro (Ș.S.); vvacaras@umfcluj.ro (V.V.); dafinm@ssnn.ro (D.F.M.); 2 “RoNeuro” Institute for Neurological Research and Diagnostic, 400364 Cluj-Napoca, Romania; gbalea10@gmail.com; 3 Department of Genomics, MEDFUTURE Institute for Biomedical Research, “Iuliu Hațieganu” University of Medicine and Pharmacy, 400349 Cluj-Napoca, Romania; 4 Department of Public Health, Babes-Bolyai University, 400294 Cluj-Napoca, Romania; razvan.m.chereches@gmail.com

**Keywords:** chronic pain, EEG, cognitive load, resting state, WM

## Abstract

Chronic pain (CP) represents a multidimensional condition in which cognitive and emotional factors shape the individual experience from perception to action. The purpose of this study was to characterize the functional significance of alterations in neural oscillatory dynamics underlying the transition from resting-state to cognitive load across distinct CP phenotypes. Continuous electroencephalographic data were acquired from patients with headache, migraine, and spine-related pain, as well as healthy controls, during rest and three visual–cognitive–motor (VCM) tasks: reaction time, working memory, and associative learning. First, within CP subgroups, we examined cognitive-load-related changes in oscillatory activity. In migraine patients, alpha/beta power attenuation induced during cognitive processing correlated with higher reported pain intensity. Relative to the spine-related pain group, migraine patients exhibited increased occipital alpha and gamma band activity during working memory and associative learning conditions, as a possible neurophysiological signature of cortical hyperexcitability. By comparing a subset of headache patients to healthy controls, we found elevated resting-state delta and gamma activity in the patient group. Under cognitive load conditions, headache patients showed higher power across delta, theta, beta, and gamma frequency bands. Delta and theta activity elicited during the working memory task correlated negatively with pain intensity. Our results demonstrate that the experience of chronic pain is accompanied by frequency-specific alterations in both resting and cognitive-associated oscillatory dynamics, reflecting impaired visual working-memory processing and top–down modulation of behaviorally relevant stimuli.

## 1. Introduction

Chronic pain (CP) requires significant resources for proper understanding, diagnosis, and treatment, as it has evolved from being considered a symptom of various disorders to a self-contained medical condition, possessing its own definition and classification [1]. The International Association for the Study of Pain (IASP) defines CP as “pain which has persisted beyond normal tissue healing time” [1,2,3]. Almost two billion people worldwide suffer from one CP subtype during their lifetime [4].

Structural changes induced by pain chronification occur at different organizational levels, ranging from molecular targets to large-scale brain networks [5]. Although the responsible processes are not entirely understood, remodeling of the spinal cord circuits is one of the first changes that occur. Maladaptive neuroplasticity lowers the nerve sensitivity threshold, while spinal circuitry shifts towards emotional and mesolimbic learning pathways [3,6,7,8]. Prolonged hyperexcitability of the nervous system can lead to central sensitization (CS), a condition characterized by abnormal modulation of both nociceptive and non-painful stimuli, resulting in higher-than-normal pain intensity and a painful perception of innocuous stimuli [6]. The most prevalent painful disorders (tension headache, back pain, and migraine) were all previously associated with a CS [9,10,11].

Cognitive and emotional factors play significant roles in the perception and integration of pain. As attention is a necessary cognitive process for environmental interaction, its mechanisms can influence CP symptomatology [12,13]. Selective attention to nociceptive signals can promote enhanced fast-band synchronization in sensorimotor areas, leading to preferential feedforward of pain-related information to downstream centers [14]. On the contrary, shifting attentional resources away from painful sensations can reduce pain intensity [13]. Retention of information during long-term memory transfer can also be hindered in CP, leading to mnemonic and learning difficulties. The enhanced attentional requirements needed to withdraw from a noxious input could also impair working memory performance. [15,16,17]. Overall, CP can compromise attention, working memory, and executive functions [15,18,19], while reports of more widespread impairment can be found in the recent literature [17,20].

Generally, cognition was evaluated in chronic pain patients (CPP) through standard instruments (e.g., clinical scales) [19], with some authors incorporating more objective methods, including electroencephalography (EEG). One of the most common and straightforward ways of interpreting EEG metrics is through neural oscillatory analysis [21,22]. Spectral profiles in CP were analyzed for various reasons, such as testing their reliability as markers of treatment efficiency [23], for machine learning classification [24], or accurate decoding of pain intensity scores derived from intracranial recordings [25].

Multiple other works analyzed the EEG spectral profile in CPP, resulting in a vast pool of findings [21,22]. While the prevalence of such initiatives remains high, oscillatory events during cognitive tasks have been little investigated, with almost no records of a spectral comparison between resting-state and cognitive effort conditions. A recent study performed a comparative evaluation of CP and major depressive disorder through microstate analysis during resting state conditions and spectral analysis during a standard-deviant paradigm [26]. At the same time, another study examined the neural oscillations of fibromyalgia patients using a resting state paired with a three-phase motor paradigm [27]. While the work of You and colleagues took into consideration cognitive states, it did not compare rest and task activity through the same method [26]. The study by Camargo et al. compared different conditions across interrelated EEG dimensions (frequency and time–frequency analysis), focusing primarily on the role of motor activity rather than cognitive effort [27].

Given the current state of literature, we aimed to analyze the cortical oscillations of a chronic pain population during the execution of three VCM tasks (relative to resting state), while looking at the resulting neurophysiological processes governing the load-dependent neural changes. In the second part of the study, we performed a unique comparison of brain activations in patients with chronic pain, relative to healthy individuals, during both resting state and cognitive load. To our knowledge, our work is the first to analyze the EEG pattern during both resting and cognitive conditions in CPP, while also looking into different pain subsets.

## 2. Materials and Methods

### 2.1. Participants

#### CP Patients

Forty-eight patients (age: 42.07 ± 13.23 [mean ± SD]), 35 females) diagnosed with CP by a neurologist were selected from a larger group of patients (n = 133) investigated at the RoNeuro Institute for Diagnostics and Research in Neurological Diseases (Cluj-Napoca, Romania) between February 2017 and February 2019 for various painful complaints. The protocol itself consisted of history taking (e.g., pain debut), a complete neurological examination with specific pain modalities/pain testing (e.g., testing pressure points for the greater occipital nerve in patients accusing cervicalgia, applying the Lasegue maneuver for presentations of low back pain, etc.), pain intensity evaluation, neuropsychological testing, EEG analysis, and eye-tracking. Every evaluated patient signed an informed consent form to participate in this study. The experimental procedure was conducted in conformity with the Declaration of Helsinki, and the study was approved by the Ethical Committee of the “Iuliu Hațieganu” University of Medicine and Pharmacy (full details can be found in the Institutional Review Board Statement section).

Inclusion requirements consisted of a valid clinical diagnosis of CP, pain intensity evaluation, pain complaints persisting for at least three months, and age > 18 at the time of the investigation. We excluded subjects diagnosed with ailments that could be associated with specific CP subtypes during their natural evolution (history of stroke or painful complications of stroke, multiple sclerosis, brain malignancies that did or did not require neurosurgical interventions) or could impair the diagnosis of CP based on responsiveness or awareness regarding symptomatology (previous comatose states/prolonged periods of altered consciousness, any form of dementia, or history of psychiatric pathologies). Patients who exhibited acute changes in CP characteristics days before examination were deemed ineligible. In addition, we did not include individuals for whom opioids or benzodiazepines were prescribed and chronically used at the moment of EEG registration, as the possible impact of the respective compounds on cerebral activity could not be controlled.

Twenty-seven patients took at least one type of pharmacological analgesic at the moment of EEG registration. Sixteen patients received antidepressants (AD): tricyclic ADs, SNRIs, and SSRIs. Fourteen patients were following one AD, while two patients used multiple agents (two, respectively, three agents of different classes). Five used only ADs for pain symptomatology management, while the other eleven used ADs together with agents from different classes. Seventeen patients followed analgesic treatment with NSAIDs, triptans, or anticonvulsants, either paired (7) or not (10) with ADs. One patient was treated with an opioid agent on an “as-needed” basis. In twenty cases, supplements, herbal products, or alternative therapies were part of the treatment scheme, with 10 patients using them alone. Eleven patients were medication-free at the time of examination (see Table 1 for complete details regarding the treatment scheme for each patient).

Two scales were applied to diagnose and quantify chronic pain: the Central Sensitization Inventory (CSI) and the McGill Pain Questionnaire (MPQ). The CSI is an instrument for disorders associated with neuronal hyperexcitability and misrepresentation of non-painful stimuli as nociceptive, both characteristics of central sensitization [28]. The MPQ evaluates the experience of pain at multiple levels (affective, evaluative, and sensory) through pain-related word sets, with the patient first selecting the most appropriate fit for his situation (e.g., throbbing, aching) and then grading it as “mild”, “moderate”, or “severe”. It, additionally, focuses on pain localization (by incorporating visuals of gross anatomical regions) and the general level of discomfort at the moment of testing [29]. Both scales were applied before the EEG recording.

The patients were distributed in three groups based on diagnosis: 28 patients with chronic headache (CH), 13 patients with migraine (MIG), and seven patients with spine-related pain (SP, comprising three patients with cervicalgia, two patients with occipital neuralgia, and two patients with low back pain, respectively). A clinically relevant central sensitization syndrome was not reached by either group (group→scores→<40). McGill scale ratings showed moderate pain intensity for CH and SP patients and moderate–severe pain for MIG patients. Complete group demographics are presented in Table 2, and patient characteristics in Table 1.

### 2.2. Healthy Controls

Twelve healthy subjects (age: 30.083 ± 8.512 [mean ± SD]), four females) were recruited to match a subgroup of headache patients for age and sex, and fulfilled the control inclusion criteria of absence of any nervous system disorders (Table 1).

### 2.3. EEG Recording Procedure

**Figure 1:**
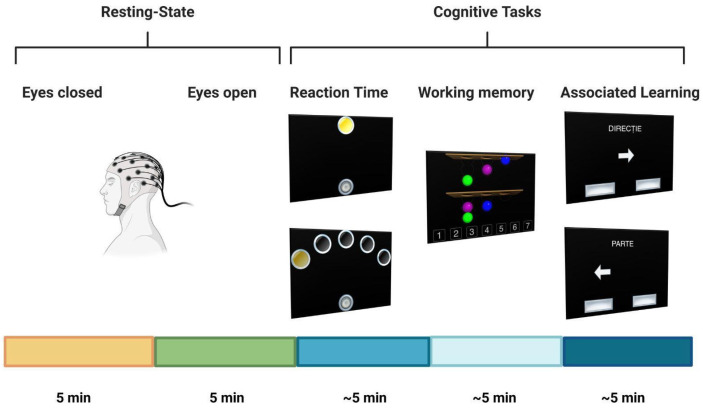
Schematic view of the experimental protocol. The data were recorded using a 32-channel EEG positioned according to the 10-10 international system during two resting-state conditions and three CANTAB^®^ cognitive tasks.

Recordings were performed in a light-dimmed room, free from external noise. Each participant was seated in an EEG armchair with integrated adjustments for lower limbs and head. Excluding the recording hardware, the room was free of electrical equipment and active artificial light sources, with only the EEG technician beside the patient. Participants were instructed to maintain proper sleep hygiene the day before the recording and avoid consuming caffeine, alcohol, energy drinks, or any products with similar effects. The recording protocol consisted of two resting state conditions (eyes open—EO, eyes closed—EC) and three cognitive load conditions, part of the Cambridge Neuropsychological Test Automated Battery assessment (CANTAB^®^, Cambridge Cognition, Bottisham, England) [30,31] (see Figure 1 for an overview of the recording protocol) Each of the tests was tailored for different cognitive dimensions (Reaction Time—RT, Working Memory—WM, and Associative Learning—AL). The technician briefly explained the protocol steps before starting the investigation and then re-explained the cognitive paradigm before the start of the first CANTAB assessment. The recording was briefly paused during the transition between tasks. A tablet running the CANTAB application was placed in front of the participant for the duration of the task-based recordings, fixed on a holder for stability. Participants underwent a training period to gain a clear understanding of the goal of each test. An individual would proceed to the actual testing period after several checkpoints of the training period were passed (mean length of 1 to 2 min), during which an audio guide and visual instructions were available. In the event of consecutive incorrect answers, instructions could be briefly re-explained only for the first test (Reaction Time), as the other two tests did not have this functionality integrated.

The CANTAB tests were used only as a mental load instrument, and their scores were not interpreted for the current study. The first test evaluated psychomotor speed through reaction time (RT) and consisted of two phases. The participant had to selectively shift between a round button placed in the lower part of the screen and one (in the first phase) or five (in the second phase) circles displayed in the upper part. The participant had to press and hold the button until a yellow dot appeared in one of the circles. Afterward, the respondent had to release the button and touch the circle that expressed the yellow dot as fast as possible, as the dot disappeared after 1 second of screen time. (See Figure 1).

The second test, called the “Stockings of Cambridge”, evaluates problem-solving capacities and working memory (annotated in this paper as WM). Participants had to mentally reshape a given object alignment based on a model configuration. Two displays were presented on the screen (model and exercise), each presenting three stockings (with the model represented in the upper display and the given exercise in the lower display). Each stocking had several slots, placed in descending order from left to right (three slots on the left, two in the middle, and one on the right). At the start of each round, three differently colored balls (red, blue, green) were randomly placed within the stockings of each display. The participant had to calculate the lowest number of movements necessary to recreate the ball configuration presented in the model without moving any of the balls in the exercise display. A numerical scale graded from 1 to 7 (representing the number of possible movements from minimum to maximum) was available at the bottom of the lower section. The participant had to select the number that is considered the solution. If the user selected a number different from the solution, a cross mark (indicating an incorrect answer) would be placed over that number until the end of the respective round. Once the participant chose the correct answer, a green check mark would appear over the respective number, and the test would proceed to the next round. The model configuration changed each round (See Figure 1).

For the third exercise, we used the Multitasking Test (MTT) to evaluate associated learning (AL) capacities. Different visual stimuli (arrows pointing to the left or the right) were presented on the screen for multiple trials. Individuals were instructed to select one of two available buttons (one left-placed and one right-placed, located near the bottom of the screen) depending on two possible cue scenarios: “pointing direction” and “arrow position”. If the word “direction” appeared to the participants, the respondent had to select the button congruent to the side towards which the arrow was pointing (e.g., if a right-pointing arrow was located in the left part of the screen, pressing the right button would be the correct answer). If the word “position” appeared on the screen, the user had to press the button situated on the same screen side as the arrow, ignoring its pointing direction (e.g., if a right-pointing arrow is located in the left part of the screen, pressing the left button would be the correct answer). Each session consisted of approximately twenty trials, with a brief pause after each session. The test gradually increased in difficulty, starting with sessions that featured a single visual cue (single-tasking) and ending with sessions that combined multiple cues (multitasking). If more than 2 seconds passed and no button was pressed for a given trial, the test would automatically jump to the subsequent one (see Figure 1).

### 2.4. Data Acquisition and Preprocessing

EEG data were recorded using 32 scalp electrodes positioned according to the 10-10 international system (Easycap, Herrsching, Germany), using Cz as the reference electrode. Impedances were kept below five kΩ. Following acquisition and storage in Nicolet (Natus Medical), each recording was exported to Brain Vision Analyzer 2.1 (Brain Products GmbH) for preprocessing. All data were further offline downsampled to 256 Hz. As the acquisition protocol involved the recording of cardiac activity through an ECG channel, data were curated from ECG artifacts, and the respective channel was removed. No EEG channels were removed from any of the recordings. Low-frequency drifts and electrical line noise were removed from the EEG data using a 1 Hz high-pass filter, a 40 Hz low-pass filter, and a 50 Hz notch filter, respectively. The trace was divided into five segments (EO, EC, RT, WM, AL) of five minutes, using the annotations placed during signal acquisition. Artifact removal was performed through independent component analysis (ICA, sphering using classic PCA, InfoMax algorithm) for both externally (electrode pop-ups and salt bridges) and internally generated (eye movements, muscle activity, and cardiac artifacts) sources [32]. The recordings were then visually inspected, with remaining artifactual items marked for removal (e.g., swallowing artifacts). Spherical splines-based topographical interpolation was applied for artifact-corrupted channels (e.g., muscular, pulse) that were either difficult or impossible to remove via ICA and manual rejection. Following offline re-referencing to a common average reference, data were exported to MATLAB (version 2025a, Mathworks, Natick, MA, USA) and analyzed using Brainstorm Toolbox [33].

### 2.5. Power Analysis

To evaluate the brain activity at different frequencies, we calculated the power spectral density (PSD) using Welch’s method [34]. For the total spectrum estimate, the signal was divided into 1 s windows, with an overlap of 50%. The Fast Fourier Transform (FFT) was computed for each segment afterward, with resulting periodograms averaged over all segments. The spectrum was split into five bands, based on default MATLAB frequency bins: delta (2–4 Hz), theta (5–7 Hz), alpha (8–12 Hz), beta (15–29 Hz), and gamma low (30–40 Hz). Spectrum normalization (1/f noise compensation) was computed by multiplying the power at each frequency bin by the corresponding frequency value.

### 2.6. Statistical Analysis

Statistical analysis was performed using JASP (version 0.19.3) [35], Brainstorm (version 3.250731), and R Studio (version 2025.05.1). For all tests, the significance level was 0.05. Data distributions were evaluated using the Shapiro–Wilk test. The Kruskal–Wallis test was utilized for comparisons among more than two groups, and the Mann–Whitney test was used for pairwise comparisons for ordinal variables. Categorical variables were contrasted using Fisher’s exact test. For each study group, we used paired permutation testing (Wilcoxon signed-rank test, two-tailed) to compare EEG power between conditions. Independent permutation testing (Student’s t, unequal variance) was applied to detect differences between patient groups and headache vs. healthy controls. Using Monte Carlo random sampling, the permutation method was repeated 1000 times and estimated the empirical distribution of the test-statistic at each EEG sensor and frequency band. The obtained *p*-values statistical map was then adjusted for multiple comparisons using the False Discovery Rate procedure (FDR) (Benjamini–Hochberg algorithm, Ntest = signals × time × frequency). Standardized effect sizes were computed for all statistical tests. For the Wilcoxon signed-rank test, we calculated the rank-biserial correlation (r_rb), with values ranging from −1 to 1. For independent permutation testing, we computed Cohen’s d by dividing the standardized mean difference between groups by their pooled standard deviation. To diminish the small-sample bias in d values, we applied Hedges’ correction (therefore, Hedges’ g is reported throughout the paper as the primary standardized mean difference between groups).

Due to recruitment constraints, in the second part of the experiment, our sample included 12 headache patients and 12 healthy controls (24 participants), and no a priori power analysis was performed. A sensitivity analysis effectuated in G*Power version 3.1.9.7 (independent samples *t*-test, two-tailed α = 0.05, 80% power) indicated a minimum detectable d ≥ 1.19, corresponding to g ≥ 1.15 after Hedges’ correction.

The correlations between clinical characteristics (pain duration, McGill score, CSI) and EEG parameters were calculated using Spearman’s correlation coefficient.

### 2.7. Task/Resting State Index

For the first experiment, the percent change in alpha and beta EEG power for each condition (Task/Resting State%) was calculated according to the equation used by Pfurtscheller [36]:

$$\;\mathrm{Task}/\mathrm{Resting-State}\;\mathrm{Index}\;=\frac{P_{task}-P_{eo}}{P_{eo}}\times \;100,$$

where *P_task_* denotes the mean power/condition and *P_eo_* is the mean resting EO power, in the respective frequency band, per participant. Positive Task/Resting State% suggests alpha/beta power decrease during the task-based period compared with the reference interval, while negative Task/Resting State% reflects alpha/beta power increase.

The degree of inter-condition alpha and beta percent power changes, as quantified by the Task/Resting-State Index, was determined using the Wilcoxon signed-rank test.

## 3. Results

### 3.1. Cognitive Oscillatory Dynamics Across Three Different Pain Subgroups

#### 3.1.1. Comparison Between VCM and EO for Each Pain Subgroup

**Figure 2:**
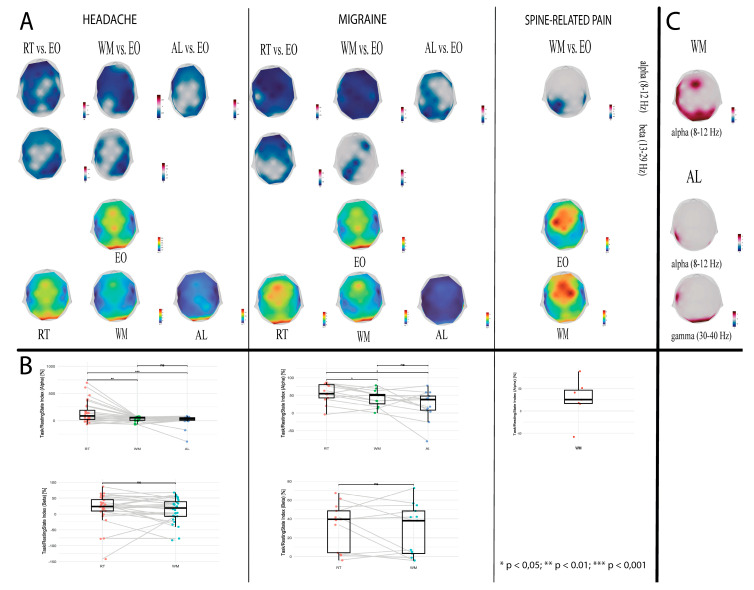
Power topographies of intragroup and intergroup differences in neural activity during the cognitive tasks, relative to resting-state, for all three pain subgroups. (**A**) Task-induced power changes and average power spectra (2–40 Hz). (**B**) Comparison of power topographies between migraine vs. spine-related pain group during the WM and AL conditions. Topographical maps depict the sensor space distribution of W and t-values. All statistical topographies were subsequent to FDR-correction of the *p*-adjusted maps obtained using the Wilcoxon signed-rank test (**left**) and Student’s *t* test (**right**). Red areas indicate power increases, blue areas reflect load-induced power decreases, gray areas display null changes. (**C**) Scatter boxplots indicating deviations in individual alpha (**top**) and beta (**bottom**) percent power change from the median value for each subgroup. Dots represent mean power across channels per frequency from each participant to illustrate inter-subject variability; significant intra-band differences between VCMs are annotated. Asterisks specify the level of significance: */**/***—*p* < 0.05/0.01/0.001.

**Figure A1:**
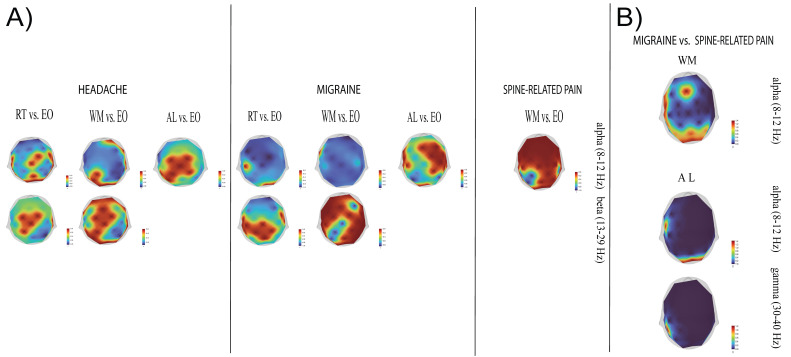
Effect size unthresholded topographical maps (rank biserial coefficient and Hedges’ g) corresponding to the contrasts displayed in Figure 2. (**A**) illustrates the magnitude of brain activity differences between conditions for each pain phenotype (RT-EO, WM-EO, AL-EO). Areas displayed in red indicate null differences between conditions, while cold colors reflect negative biserial rank correlations (indicating greater activity in EO relative to the comparison condition). (**B**) displays the corresponding effect size maps for group-level comparisons between migraine vs. spine-related pain patients. Cortical areas displayed in red indicate the largest effect sizes, and blue areas correlate with null differences between groups.

Compared to the resting state, cognitive load produced alpha and beta band desynchronization in multiple brain areas (after multiple comparison correction) (Figure 2A, upper panel for all subgroups and conditions, Appendix A Figure A1A).

**Headache group.** During the RT condition, alpha attenuation was observed in thebilateral orbitofrontal, dorsolateral prefrontal, and inferior frontal regions, extending to the left premotor, supplementary motor, primary motor, somatosensory, supramarginal, and superior parietal areas(Fp1/2, Fz, F3/F4, F7/F8, FC1, C3/4, CP1/2, CP5/6, P3/4 electrodes) The most substantial effect was observed at Fp1 (W_min = −346, r_rb = −0.85), with effect sizes across significant electrodes ranging from −0.45 to −0.85. Beta activity desynchronization during RT was more widespread, encompassing the bilateral orbitofrontal, dorsolateral prefrontal, inferior frontal, and sensorimotor cortices, as well as the superior temporal and parietal (including the precuneus and supramarginal gyrus) areas,, left temporo-occipital and visual areas(Fp1/2, Fz, F3/4, F7/8, C3/4, FC5/6, C3/4, T7/8, P7/8, CP2, CP5/6, Pz, P3/4, PO9, Oz, O1/2 electrodes; r_rb range: −0.44 to −0.83).

During the WM task, alpha activity reductions were localized to the orbitofrontal, and dorsolateral prefrontal areas, left inferior frontal (including opercular cortex), motor, supramarginal, and, superior parietal regions, the precuneus, and the fusiform areas (Fp1, Fz, F3/4, F7, C3/4, Cz, FC1/2, FC5/6, T7/8, Pz, P4, TP9; maximal at CP6: W_min= −352, r_rb = −0.86). Beta power attenuation involved the bilateral dorsolateral prefrontal cortices, inferior frontal, primary and sensorimotor areas, the superior temporal, supramarginal, superior parietal regions, and the precuneus (F3/4, FC5/6, C3/4, T7, CP2, CP6, Pz, P4; W_min= −292 at P4 electrode, r_rb = −0.71).

In the AL task, alpha activity attenuation extended across the orbitofrontal, dorsolateral prefrontal, inferior frontal, sensorimotor, temporal, parietal (including precuneus), temporo-occipital (including the fusiform area), and visual regions, (Fp1/2, Fz, F3/4, F7/8, FC5/6, C3/4, T7/8, P7/8, PO9/10, TP9/10, Oz, O1/2, Pz, P4, CP6; maximal at F7: W_min= −332, r_rb = −0.81).

**Migraine group**. The RT condition elicited alpha and beta activity attenuation across the frontal, motor, temporal, parietal, and occipital cortices. Alpha band decreases were maximal at F3 and FC2 (W_{min} = −91, r_{rb} = −1.0), with stronger anterior than posterior effects. Beta attenuation was widespread, peaking at Fp2 (W_{min} = −91, r_{rb} = −1.0).

During the WM task, alpha power attenuation extended across the bilateral orbitofrontal, dorsolateral prefrontal, and inferior frontal (including theopercular cortex) regions, as well as the sensorimotor, superior temporal, parietal (including precuneus and supramarginal gyrus), temporo-occipital (including fusiform area), and visual cortices (Fp1/2, Fz, F3/4, F8, FC1/2, FC5/6, Cz, C3/4, FC5/6, T7, P7, CP1/2, CP5/6, Pz, P3/4, PO9/10, TP9/10, Oz, O1/2 electrodes; Fp2: W_min = −89 r_rb = −0.97). Beta activity decreases were more focal, involving the right dorsolateral prefrontal cortex, sensorimotor cortices, left superior parietal lobule, and the precuneus (F4, Cz, CP1, P3 electrodes; W_min = −79, r_rb_min = −0.86 at F4 electrode).

During the AL task, alpha activity attenuation was observed in the orbitofrontal, dorsolateral, and inferior frontal cortices, sensorimotor cortices, parietal (including the precuneus and supramarginal gyrus), and the visual regions (Fp1/2, F3, Pz, P3/4, P7/8, F8, F3, FC1, Cz, CP1, CP5, O2; P3: W_min = −91, r_rb = −1).

**Spine group**. Cognitive-load-associated changes were more restricted compared to the other groups. Significant alpha band attenuations were observed during the WM task at the level of the bilateral supramarginal gyri, left postcentral gyrus, left superior parietal lobule, and the precuneus (electrodes; r_rb range −0.85 to −1 across significant electrodes: CP1, CP5/6, Pz, and P3). No significant beta activity effects were found.

#### 3.1.2. Comparison of Power Changes Produced Under Cognitive Load Between Pain Subtypes

We found significant differences in brain activity associated with the transition to working memory load between the migraine and spine-related pain groups (Figure 2C and Figure A1B). During the WM task, migraine patients exhibited stronger alpha activity relative to the spine group across frontal (middle and left inferior frontal gyri), temporal (left superior temporal cortex), parietal (bilateral superior parietal lobule, precuneus, left supramarginal gyrus), and occipital regions (maximal at Fz:, t(18)_max = 3.11, Hedges’ g = 1.42. Between-group contrasts revealed further alpha differences during the AL task at the level of the left inferior frontal gyrus and bilateral occipital cortices (FC5, Oz, O1/2 electrodes; maximal at FC5: t(18)_max = 3.2 g = 1.44). In the gamma band, migraine patients showed increased activity in the left supramarginal gyrus and right inferior temporal cortex (CP5: t(18) = 2.73, g = 1.22, PO10: t(18) = 1.71, g = 0.77). No significant differences between migraine and spine-related pain groups were registered during the resting state.

#### 3.1.3. Task/Resting-State Index

The analysis of alpha power percent change between cognitive load and resting-state revealed significantly lower alpha values during the first condition, with differences between RT and WM tasks (*p* < 0.01) and RT and AL tasks (*p* < 0.001) in the headache group. Additionally, comparable contrasts were observed in the migraine group, with higher alpha percent change values during RT, relative to both WM (*p* < 0.05) and AL tasks (*p* < 0.05). By contrast, beta power did not show significant variability across conditions (Figure 2B).

### 3.2. Global Brain Activity Contrasts Between Headache and Control Groups

#### 3.2.1. Comparison of Power Between VCMs and EO in the Headache and Control Group

**Figure 3:**
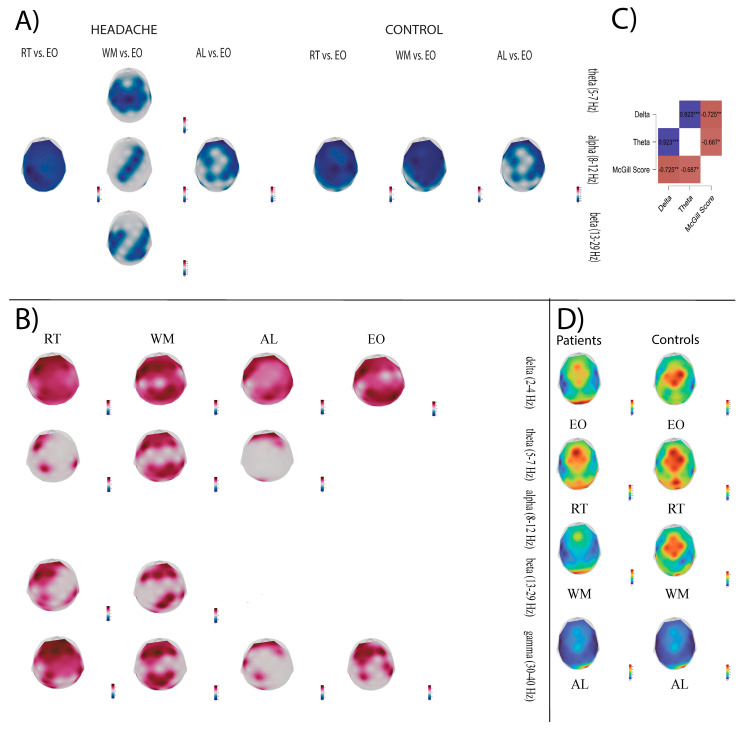
Power topographies of intragroup task-induced changes (all conditions relative to RS) and intergroup (headache vs. control) changes during RS and VCM. (**A**) Task-induced power in the patient group (**left**) and controls (**right**). (**B**) Power differences between patients and controls at delta, theta, beta, and gamma frequencies. Topographical maps depict the sensor space distribution of W and t-values. All statistical topographies followed FDR-correction of the *p*-adjusted maps obtained using the Wilcoxon signed-rank test (**top**) and Student’s t test (**bottom left**). Red areas indicate power increases, blue areas reflect power decreases, and gray areas display areas with null changes. (**C**) Heatmap displaying significant correlations during the WM task in the headache group. Asterisks specify the level of significance: */**/***—*p* < 0.05/0.01/0.001. (**D**) Average power spectra (2–40 Hz) for patients (left) and controls (right).

**Figure A2:**
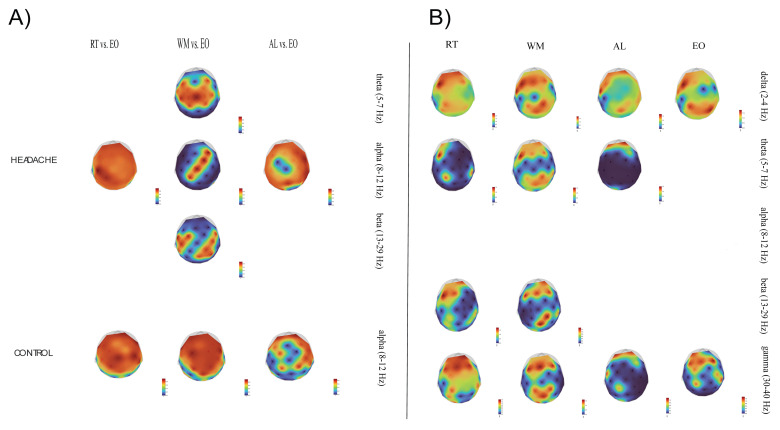
Effect size unthresholded topographical maps (rank biserial coefficient and Hedges’ g) corresponding to the contrasts displayed in Figure 3. (**A**) illustrates the magnitude of brain activity differences between conditions for each group (upper rows—patients, lower rows—healthy controls). Cortical areas displayed in red indicate greater activation in the comparison condition compared to EO, and blue regions correspond to null differences between conditions. (**B**) displays unthresholded Hedges’ g maps, indexing the contrasts between groups (headache patients vs. controls) for each frequency band. Cortical areas displayed in red indicate the largest effect sizes, and blue areas correlate with null differences between groups.

After FDR correction, both groups showed significant alpha power decreases during the transition from RS to the VCMs (Figure 3A shows FDR-corrected power differences between conditions, Figure A2A displays the corresponding effect sizes—Hedges’ g).

**Headache group.** In the RT—EO transition, alpha power attenuation was global, with maximal decreases in the left sensory–motor region (C3: W = −76, r_rb = −0.97, CP1: W = −72, r_rb = −0.92). The comparison of WM vs. EO indicated theta decreases in the frontal–central–parietal regions (maximal at Cz electrode: W = −78, r_rb = −1). At the same time, alpha power was reduced in the right dorsolateral prefrontal, somatomotor, and right parieto-occipital cortices, with large effects (r_rb = −0.60 to −0.94 across significant sites). Beta power also decreased in the bilateral sensorimotor cortices (FC1, FC5/6, C3/4, CP2, CP5, Pz, P4, maximal difference at FC5: r_rb = −0.92). In the AL—EO transition, a diffuse alpha power attenuation was observed, with the strongest effects at the level of right inferior frontal, medial parietal, and right parieto-temporal regions (FC6, Pz, CP6, P8, TP10, r_rb range: −0.64 to −0.92).

**Control group.** RT—EO transition revealed global alpha attenuation, strongest in the bilateral inferior frontal, right orbitofrontal, right motor, left somatosensory, and right supramarginal areas (FP2, F7, F8, Cz, C4, CP1, CP6, r_rb range: −0.66 to −0.94). WM—EO transition produced alpha decreases in the bilateral inferior frontal and left somatomotor regions (F7, FC6, FC1, CP1, r_rb range: −0.66 to −0.94). AL vs. EO, indicated alpha reductions across the orbitofrontal, dorsolateral, inferior frontal, bilateral somatosensory, temporal, right motor, left premotor, and extrastriate cortices (Fp1/2, F3/4, F7/8, C4, FC1, FC6, CP1, CP6, P7, r_rb range: −0.64 to −0.89).

#### 3.2.2. Brain Activity Comparison Between Headache and Control Group

During both RS and the VCMs, the patient group demonstrated significantly greater brain activity than controls across several frequency bands (Figure 3B and Figure A2B).

**Resting state.**

Group differences were observed at the level of the frontoparietal cortex in delta (maximal differences at F3: t(22) = 4.59, g = 1.81, and P4: t(22) = 4.46, g = 1.75) and gamma frequency bands (t(22)_max = 3.43, g = 1.35 at F4 electrode).

**Delta band during VCMs.**

During the RT task, delta activity was significantly higher in the fronto-centro-parietal regions (t(22)_max = 6.30 at F3, g = 2.48 during RT). In WM and AL tasks, similar regional power differences were observed, with maximal effects at the level of FC5 electrode (t(22)_max = 4.65, g = 1.83, respectively, t(22)_ max = 4.21, g = 1.74).

**Theta band during VCMs.**

Headache patients presented higher theta power during RT in the orbitofrontal, dorsolateral prefrontal, and somatomotor cortices (Fp1, F3, CP1, CP6, t(22)_max = 3.85, g = 1.52 at F3). During WM, theta activity power differences encompassed the bilateral frontal and parieto-occipital cortices (Fp1/Fp2, F3/F4, Fz, CP1/2, CP6, P3/4, O1/2, t(22)_max = 3.94, g = 1.55 at F3. During AL, positive power differences were observed across the bilateral orbitofrontal and left occipital cortices (Fp1/2, O1, t(22)_max = 3.80, g = 1.50 at Fp2 electrode).

**Beta band during VCMs.**

During RT, power differences were registered in the orbitofrontal, dorsolateral prefrontal, medial superior frontal, inferior frontal, premotor, and parietal regions (Fp1/2, F3, Fz, FC1, FC5, CP1, CP6, P3/4, t(22)_max = 4.57, g = 1.80 at F3 electrode). During WM transition, beta activity differences were present in the orbitofrontal, dorsolateral prefrontal, medial frontal, somatosensory, supramarginal, posterior parietal, and occipital regions (Fp2, F3/4, Fz, Pz, CP2, CP6, O1, t(22)_max = 3.45, g = 1.36 at CP2 and Pz).

**Gamma band during VCMs.**

RT transition was accompanied by greater gamma power in the fronto-centro-parietal, inferior temporal, and occipital cortices (Fp1/2, Fz, F3/4, FC1/2, Cz, CP1, CP6, P3, P8, O1, TP9/10, t(22)_max = 3.64, g = 1.43 electrode). WM transition was associated with positive power differences in the bilateral frontal and parieto-occipital cortices during WM (Fp1/2, F3/4, Fz, FC1, CP1/2, Pz, P3, O1/2), t(22)_max = 4.05, g = 1.64 at Fz electrode). During AL, gamma activity was increased in the bilateral orbitofrontal, right dorsolateral prefrontal, left inferior frontal, and left somatosensory cortices (Fp1/2, F4, FC5, CP1, t(22)_max = 2.98, g = 1.17 at Fp1).

No differences between groups were observed in the alpha frequency range.

### 3.3. Relationship Between Cortical Oscillatory Dynamics and Clinical Features

We restricted our analysis to significant values between conditions and groups to minimize the number of statistical tests. We calculated the correlations between mean alpha and beta power across sensors and clinical features, pain duration and intensity, for the first part of the study. Overall, we found a strong entrainment of alpha and beta activity across conditions (ρ=0.563–0.929), accompanied by a significant positive correlation between McGill scores and CSI (ρ= 0.504). Additionally, headache patients presented an inverse relationship between the McGill score and disease duration, suggesting increased pain intensity closer to disease debut (ρ=−0.382) In the migraine group, alpha and beta band activity during the execution of the RT (alpha: ρ = 0.657, beta: ρ = 0.566) and WM (alpha: ρ = 0.687, beta: ρ = 659) conditions correlated positively with the McGill score, while during AL only the beta band reached statistical significance (ρ = 0.582) (see Table 3). In the second part of the study, correlations were computed between the mean power across channels for all five frequency bands during RS/VCM and clinical data for the patient group, respectively. Only the WM condition presented strong negative correlations between delta, respectively, theta band power, and the McGill score (delta: ρ = −0.725, theta: ρ = −0.687), see Figure 3C.

## 4. Discussion

In this study, we attempted to evaluate whether neural oscillatory activity is objectified in different ways among patients with various chronic pain phenotypes who are undergoing three types of VCM protocols. The purpose of this comparative study was to examine the processes underlying task–rest transitions and provide insights into possible altered neural dynamics accompanying these shifts.

Alpha and beta power attenuations were observed in the brain regions involved during cognitive processing in response to task demands (the equivalent of the event-related desynchronization phenomenon from event-related studies). The exception to this pattern was represented by the spine-related pain group, which showed power changes restricted to the alpha band (most likely because of a small sample size).

Self-paced finger movements produced alpha and beta band power changes (predominantly alpha) at the level of the primary somatosensory cortices and supplementary motor areas. Alpha/beta oscillatory suppression was present in the prefrontal and parietal cortices during all tasks in the headache and migraine groups. In EEG-fMRI paradigms, the fMRI BOLD signal at the level of the dorsolateral prefrontal cortex correlates negatively with low-frequency activity (alpha and beta), thereby mediating accurate performance during encoding and maintenance periods in spatial working memory tasks [37]. Displaying some contrasts in temporal parameters, the neural activity of the prefrontal and parietal cortices occurs congruently in working memory tasks [38], as present in our findings. Based on inherent properties (spatial/non-spatial) in processing external objects, their circuitries dichotomize into different functional modules. The ventral stream (“what” pathway) encodes the external personal space for grasping movements, visual object configurations, and is implicated in visually paced finger manipulation, through anatomical connections between the bilateral inferior frontal gyrus and bilateral intraparietal sulcus. In the other frontoparietal circuit (dorsal visual stream, “where” pathway), the bilateral superior frontal gyrus displays task-based co-activation with the superior parietal lobule and bilateral inferior parietal lobules (via the superior longitudinal fasciculus), owing to a spatial code implicated in action-related processes (saccades, fixation, action execution/inhibition, sequence learning, etc.) [39,40]. Previous studies demonstrated the role of alpha/beta activity decreases in the parieto-occipital areas with the allocation of top–down visuo-spatial attention and the active maintenance of the features associated with the current sensorimotor set. Furthermore, alpha/beta activity attenuation within the superior temporal cortex denotes the processing of linkages between concurrent visual stimuli [41].

However, movement-related oscillatory changes during the execution of simple motor tasks (in the absence of cognitive manipulation) were previously reported at the level of extra-motor areas, namely orbitofrontal, dorsolateral prefrontal, premotor, cingulate, mesial and lateral temporal, and inferior parietal cortices, intervening in on-task time perception (via connections with the striatum) and as memory companions of repetitive movement [42].

Generally, we found spatially diffuse desynchronization over the frontal and parieto-occipital areas (and to a smaller extent over the sensorimotor cortex), across all investigated subjects during the experiments in the alpha and beta bands. This pattern of cortical activation reflects not only the working memory load or difficulty of the task, but similarly task-on-task performance and resource allocation [43,44]. Indeed, our data showed that alpha and beta activity attenuation correlated with pain intensity as indexed by the McGill score in the migraine group (which displayed moderate–severe pain ratings).

Additionally, our findings demonstrate that headache and migraine patients displayed greater attenuation of alpha-oscillations during the execution of RT compared with WM and AL tasks (Figure 2B). Based on the topographical power changes reflecting a widespread dispersion, the role of lower-alpha oscillations (8–10 Hz) involved in attentional and top–down control mechanisms, gating WM information, can be sustained. Subsequently, both groups displayed less alpha attenuation, suggesting a shift towards the inhibition of task-irrelevant contents interfering with WM representations [45,46]. Previous results demonstrated that load-dependent alpha modulation correlates with behavioral performance and scales with the number and complexity of items held in WM [47].

By comparing inter-group differences in VCM processing, we observed greater oscillatory activity in alpha and gamma frequency bands in migraine patients compared with the spine-related pain group. Alpha-power increases were located over cortical networks of the bilateral medial, left inferior frontal, left temporal, left supramarginal, parietal, and occipital regions, while enhanced gamma-band activity was observed over the bilateral temporal, parietal, and occipital cortices. This differential task-induced power increase can be partly attributed to the state of generalized neuronal hyperexcitability characteristic of migraine pathophysiology, determined by an altered excitatory–inhibitory balance. Some studies suggest that at the molecular level, patients with more severe and long-lasting forms of migraine present lower GABA concentrations in the occipital, anterior cingulate, and medial prefrontal cortex, as determined by magnetic resonance spectroscopy estimations. In turn, reduced GABA levels increase alpha-band power, while benzodiazepines are known to inhibit it [48,49,50,51]. Moreover, in an fMRI study, visual stimulation produced a lateralized visual network activation. Hence, it can be hypothesized that these interhemispheric differences contribute to visual aura symptoms and further propagate along the dorsal and ventral visual streams [52,53].

Compared with the spine-related pain group, other locations in which alpha power activation was found for migraine patients included the medial prefrontal cortex, bilateral superior parietal lobules, the precuneus, and the left supramarginal gyrus, which are constituents of the “pain matrix” and major contributors to chronic migraine symptoms [54]. Lastly, we registered increased alpha activity over the left inferior frontal and superior temporal cortices. Connectivity alterations were previously reported at these levels in migraine patients, reflecting impairments in multisensory integration producing language disabilities [55]. Indeed, in studies using temporal discrimination protocols, higher alpha synchronization (usually, parieto-occipital) leads to lower inter-modality sensory perception [56].

Further, we aimed to assess the resting-state and task-induced brain activity differences between a subset of the headache group and healthy controls. The patient group exhibited enhanced and widespread activity in the delta band and increased gamma power in the frontal, central, and right parietal regions during the resting state. Relative to controls, cognitive load produced global delta power enhancement and complex, mixed theta, beta, and gamma power increases. Different studies previously reported the relationship between delta oscillations and chronic pain at rest, suggesting their importance at the level of perception and for the compensatory mechanisms that distract the focus of attention away from the experience of pain [57,58]. In the cognitive domain, delta activity enforces an inhibitory function over the emotional and motivational states under the control of the prefrontal cortex and participates in suppressing task-irrelevant sensory input [59,60]. Our findings parallel past evidence, as the most robust differences for the patient group were found in the delta band during the reaction time task.

During the second task, we found an inverse relationship between the delta and theta s power levels and pain intensity, as measured with the McGill score. In line with the similar gating mechanism provided by f(mid)frontal theta oscillations upon distracting representations in WM processes [61,62], our observation suggests that an increased pain perception negatively influences the autonomy to regulate the endogenous mechanism of selective attention. In healthy individuals, goal-directed attention (e.g., the Stroop task) and mind wandering produce sustained antinociceptive modulation [63,64]. Attentional bias malleability, however, constitutes an essential factor enforcing the chronification of disability and pain [65].

Relative to healthy controls, the patient group exhibited increased theta power in the frontal region during all cognitive tasks. In contrast, theta power increases in the parieto-occipital and left temporal regions accompanied the WM task. As frontal theta mechanisms participate in attentional control, research suggests that the posterior generators of theta oscillations (located in temporal and occipital cortical areas) facilitate memory encoding and retention [66].

Beta oscillations were enhanced in the patient group at the level of the frontoparietal network in the first two tasks. The exacerbation of beta band activity reflects primarily a functional inhibition filter of action and cognition (manifested especially in Parkinson’s disease) [67]. Enhanced frontal beta activity was observed following unexpected perceptual events and during error processing, leading to post-error motor slowing and decreased reaction time on the subsequent trials (via subthalamic nucleus beta activity and catecholaminergic/dopaminergic modulation of arousal) [68]. Furthermore, frontoparietal beta oscillations are the main rhythmic components of the dorsal attentional stream, involved in saccade generation and perception-guided behavior [69]. In a previous EEG resting-state paradigm, prefrontal beta and gamma oscillations were associated with the subjective evaluation of pain [70].

Lastly, we observed increased gamma activity in the visual, dorsal, and ventral cortices during all cognitive conditions. Enhanced gamma frequency synchronization promotes the encoding of relevant behavioral stimuli in sensory cortices, along with further WM representation maintenance and feed-forward transmission through the downstream areas. Feedback interactions, such as dorsal visual to early visual and dorsal visual to ventral visual, operate in the alpha/beta frequency range, scaling with WM demands. A method to analyze the neuronal synchrony would be needed to generalize our observations, such as coherence [71,72].

At the level of task-induced oscillatory changes, alpha band attenuation was present in both the headache and control groups. During the WM task, the headache group presented theta power attenuations, additional to alpha/beta decreases. The most consistent theta signature in working memory tasks appears at the level of the frontal–midline cortex and correlates with higher behavioral performance. Surprisingly, we did not find load-induced midline power changes, but bilateral dlPFC and sensory–motor theta power decreases relative to the resting-state. Load-related theta power decreases were previously observed in intracranial recordings of epilepsy patients at the level of the dlPFC [73]. A possible mechanism at play may involve the cross-frequency coupling between frontal low-frequency activity (delta–theta) and posterior alpha “gating” activity, facilitating top–down goal-dependent prioritization of information processing within visual WM [74].

A number of limitations need to be addressed as well. First of all, low-density EEG recordings preclude accurate source localization, so our analysis was restricted solely to sensor-level analysis. Furthermore, Zelmann and colleagues cautioned that a satisfactory sampling of high-frequency oscillations should involve electrode configurations with higher density than used in our experiment [75]. Considering the overlap between fast cortical activity and muscular artifacts at frequencies in the high beta range and above, we filtered the data through semi-manual ICA [76,77]. Although the recordings were carefully cleaned using both ICA and manual artifact rejection, we cannot rule out the possible incidental elimination of high-frequency cortical oscillations. As psychometric task results during cognitive tasks were unavailable for patient evaluation, no correlation between electrophysiological data and behavioral scores could be obtained.

Hormonal imbalance could be one of the agents behind gender divergence regarding CP, especially in women who express high estrogen and low testosterone levels. The resting-state periodic and aperiodic components of brain activity, as well as visuospatial performance, are known to be stable in women across the estrus cycle. However, evidence supports the idea that men have superior visuospatial abilities and perform better in tasks requiring top–down attentional control [78,79,80]. These neurobiological differences can be further coupled with the observed anti-inflammatory effect of elevated testosterone in men, supporting different nociception phenotypes [81]. Considering these facts, future studies should aim to better elucidate the influence of gender on CP oscillatory activity and explore whether this influence varies between chronic pain subpopulations.

Although the present study demonstrated differences in brain oscillatory activity across pain phenotypes and VCM conditions, the sample sizes differ between the three patient groups (with the spine-related pain group being markedly underrepresented). Moreover, sample size limitations for each group increase the risk of Type II errors.

Due to recruitment constraints, the sex distribution differs between the patient-only and patient–control comparisons. In the first part of the study, the chronic pain participants were predominantly females, reflecting the higher prevalence of headache and migraine in this population. However, in the second part of the study, where a subset of headache patients was compared with healthy controls, females represented about one-third of the total number of participants. This limitation may prevent the generalizability of our results, particularly when extrapolating to more balanced datasets.

Another limitation resides in the heterogeneous therapeutic schemes that most of the patients were following, as we found it difficult to control the possible effect of each drug or intervention. Twenty-two (45.83%) patients out of the entire population received centrally acting agents (CAA, 16 out of 22 using antidepressants). Recent evidence regarding the role of antidepressants in chronic pain highlights the beneficial effect of Duloxetine (SNRIs) for the ease of pain and reducing its intensity, with small to moderate effects. However, Duloxetine was the only agent out of 25 examined for which the authors state their certainty regarding its efficacy in chronic pain treatment [82]. Similar reports [83,84] strengthen the beneficial role of Duloxetine for various painful conditions, including knee osteoarthritis [83], back pain [83], or neuropathic pain [84]. However, while most studies evaluating the role of ADs in chronic pain excluded headache trials, the work in [84] describes the beneficial role of tricyclic antidepressants (e.g., amitriptyline) in chronic tension-type headache. In our population, two patients used Duloxetine in their scheme, both diagnosed with tension-type headache, while 15 received amitriptyline (10 diagnosed with tension-type headache), by far the largest antidepressant drug prescribed. Another recent work assessing the role of CAA on two chronic pain cohorts through multiple layers (peak alpha frequency, network measures, and oscillatory power) of analysis found evidence against the effect of studied CAA (antidepressants, antiepileptic, or opioids) on EEG features [85]. While a separate analysis regarding treatment effects would have been beneficial, we did not consider it for the current paper.

Although our study permitted us to unravel the brain dynamics involved in a limited spectrum of cognitive processes at different frequency bands, we are cautious about further generalization, as we did not perform source-based reconstruction. Moreover, the most robust cognitive-induced neural alterations associated with chronic pain were present in the delta (during all conditions), beta (RT task), and gamma (WM task) bands. Of these, only working-memory-associated delta activity correlated negatively with pain intensity. According to our study, CP patients have altered working memory processing due to impaired top–down control, deficient gating mechanisms, and attentional filtering of relevant stimuli.

## Figures and Tables

**Table 1:** Patients’ clinical profile.

Patients	McGill Score	CSI Score	Age	Sex	Chronic Pain Type	Medication Class/Therapeutic Approach	Risk Behaviors/Factors for Pain	Pain Duration (Months)
**1**	14	40	54	F	Tension-type headache	Supplements/Herbal Remedies	Smoker Prolonged Standing	60
**2**	25	9	44	M	Tension-type headache	Supplements/Herbal Remedies Acupuncture	Occasional Alcohol Consumption	300
**3**	20	26	48	F	Tension-type headache	Homeopathy	Smoker Occasional Alcohol Consumption	90
**4**	30	19	31	F	Migraine without aura	None	Overweight	60
**5**	23	37	43	F	Tension-type headache	Tricyclic Antidepressants	Smoker	180
**6**	16	59	52	F	Tension-type headache	Tricyclic Antidepressants Analgesics	None declared	66
**7**	42	43	31	M	Tension-type headache	Tricyclic Antidepressants SNRIs SSRIs Other Analgesics	Smoker	131
**8**	15	50	68	F	Migraine (possibly with aura)	None	None declared	108
**9**	40	25	50	F	Migraine without aura	Tricyclic Antidepressants Supplements/Herbal Remedies	None declared	204
**10**	25	52	44	M	Cluster-type Headache	Supplements/Herbal Remedies	Smoker Occasional alcohol consumption	6
**11**	10	21	43	F	Arnold Neuralgia	Supplements/Herbal Remedies Tricyclic Antidepressants	None declared	3
**12**	26	31	24	M	Tension-type headache	Ergot Derivates Supplements/Herbal Remedies Tricyclic Antidepressants Other Analgesics	None declared	4
**13**	33	56	44	F	Tension-type headache	Supplements/Herbal Remedies Tricyclic Antidepressants Anticonvulsants	None declared	60
**14**	32	36	31	F	Transformed Migraine	Triptans Tricyclic Antidepressants Other Analgesics	Analgetic Abuse	6
**15**	20	42	30	F	Tension-type headache	NSAIDs Supplements/Herbal Remedies	None declared	0.75
**16**	30	37	29	F	Tension-type headache	Supplements/Herbal Remedies Tricyclic Antidepressants	None declared	18
**17**	7	38	68	F	S1 Radiculopathy Dorsal Spondylosis Cervical C4-C7 disk protrusions	Supplements/Herbal Remedies	None declared	48
**18**	10	18	49	F	Tension-type headache	NSAIDs Supplements/Herbal Remedies	None declared	60
**19**	10	33	47	F	Degenerative C5-C6 disk hernia	N/A	N/A	12
**20**	20	23	33	F	Transformed Migraine	Supplements/Herbal Remedies	None declared	6
**21**	28	30	35	F	Migraine without aura	Tricyclic Antidepressants	Smoker	24
**22**	5	2	25	M	Tension-type headache	NSAIDs	Smoker Energy Drinks Psychoactive Drugs	48
**23**	16	40	48	M	Tension-type headache	Supplements/Herbal Remedies Tricyclic Antidepressants	None declared	1
**24**	55	36	47	F	Migraine without aura	Triptans Tricyclic Antidepressants	Smoker	396
**25**	18	58	42	M	Tension-type headache	Tricyclic Antidepressants SSRIs	Recent suppression of caffeine excess Recent suppression of smoking	18
**26**	39	19	69	F	Arnold Neuralgia	Anticonvulsivants	None declared	36
**27**	32	61	35	F	Tension-type headache	Tricyclic Antidepressants	Smoker Night Shifts	12
**28**	40	62	23	F	Migraine	NSAIDs	None declared	60
**29**	36	71	70	F	Tension-type headache	Supplements/Herbal Remedies	Obesity	36
**30**	37	63	35	M	Degenerative C7-C8 disk disease	NSAIDs	None declared	12
**31**	14	35	51	M	Tension-type headache	Tricyclic Antidepressants Anticonvulsivants	None declared	132
**32**	41	73	41	F	Tension-type headache	None	Night Shifts	60
**33**	26	34	34	F	Mixed tension headache Pharmacologically induced Headache (Multiclass drug-resistant pain)	Triptans Non-NSAID analgesics	None declared	96
**34**	27	34	60	F	Tension-type headache with migraine elements	Supplements/Herbal Remedies Non-NSAID analgesics	Occasional alcohol consumption Occasional smoking	180
**35**	25	46	51	F	Migraine and Hypnic Headache	NSAIDs Supplements/Herbal Remedies Triptans	Emotional Distress	120
**36**	41	46	38	M	Tension-type headache	Opioids (Multiclass drug-resistant pain)	None declared	36
**37**	34	33	72	F	Degenerative C3-C6 disk disease	None	None declared	60
**38**	9	27	34	F	Tension-type headache	None	None declared	60
**39**	10	30	28	F	Migraine	Supplements/Herbal Remedies (Multiclass drug-resistant pain)	None declared	42
**40**	20	12	41	F	Tension-type headache	SNRIs	None declared	168
**41**	51	47	36	F	Transformed Migraine	Triptans	Analgetic abuse	264
**42**	13	12	24	M	Low Back Pain	None	None declared	60
**43**	28	35	32	M	Migraine with aura	None	None declared	214
**44**	27	34	30	F	Tension-type headache	None	Smoker Alcohol consumption	12
**45**	5	1	63	M	Tension-type headache	Supplements/Herbal Remedies	Alcohol consumption	36
**46**	18	10	37	M	Tension-type headache	None	None declared	1
**47**	39	33	46	M	Chronic headache	None	Emotional Distress	516
**48**	32	58	24	M	Migraine with aura	Supplements/Herbal Remedies	Smoker Alcohol consumption	18

N/A—not available; NSAIDs—nonsteroidal anti-inflammatory drugs; SNRIs—Serotonin–norepinephrine reuptake inhibitors; SSRIs—Selective serotonin reuptake inhibitors.

**Table 2:** Group Demographics.

				Shapiro–Wilk Test	Significance Testing (Statistical Test)			Shapiro–Wilk Test	Significance Testing (Statistical Test/Mean)
Condition	Headache (n = 28)	Migraine (n = 13)	Spine-Related Pain (n = 7)			Headache Subgroup (n = 12)	Control (n = 12)		
**Age (Mean** **± SD)**	42.393 ± 11.272	37.615 ± 12.862	51.143 ± 18.792	Headache—0.651; Migraine—0.075; Spine—0.349	0.168 (Kruskal–Wallis Test) *	34.333 ± 7.475	30.083 ± 8.512	Headache Subgroup—0.364 Control—0.010	<0.164 (Mann–Whitney U test)
**Sex (M/F)**	12/16	2/11	2/5	N/A	Headache vs. Migraine = 0.156; Headache vs. Spine = 0.676; Migraine vs. Spine = 0.587 (Fisher’s Exact Test, all three comparisons)	8/4	8/4	N/A	1.00 (Fisher’s exact test)
**McGill Score (Mean** ± **SD)**	23.500 ± 10.655	31.231 ± 12.950	21.429 ± 14.432	Headache—0.492; Migraine—0.863; Spine—0.036	0.163 (Kruskal–Wallis Test)	27.000 ± 10.436	N/A	N/A	N/A
**CSI (Mean** ± **SD)**	36.464 ± 19.259	38.231 ± 13.430	31.286 ± 16.760	Headache—0.554; Migraine—0.650; Spine—0.418	0.608 (Kruskal–Wallis Test) *	31.083 ± 15.826	N/A	N/A	N/A
**Average Pain Duration (Months, Mean** ± **SD)**	85.741 ± 110.125	119.077 ± 121.586	33 ± 24.062	Headache—0.001; Migraine—0.037; Spine—0.492	0.225 (Kruskal–Wallis Test)	96.896 ± 157.821	N/A	N/A	N/A

* While normality was confirmed for some variables, applying a parametric test could lead to decreased sensitivity due to the small sample size of the Spine-related pain group. CSI—central sensitization index; F—female; M—male; N/A—not appliable; SD—standard deviation.

**Table 3:** Correlations Between Clinical Parameters and Alpha/Beta Band Activity.

	Headache					Migraine					Spine-Related Pain				
Reaction Time	Alpha	Beta	CSI Score	McGill Score	Pain Duration (months)	Alpha	Beta	CSI Score	McGill Score	Pain Duration (months)	Alpha	Beta	CSI Score	McGill Score	Pain Duration (months)
Alpha		0.672 ***					0.753 **		0.657 *						
Beta	0.672 ***					0.753 **			0.566 *						
CSI Score				0.504 **											
McGill Score			0.504 **		−0.382 *	0.657 *	0.566 *								
Pain Duration (months)				0.382 *											
**Working Memory**															
Alpha		0.588 **					0.802 **		0.687 **			0.857 *			
Beta	0.588 **					0.802 **			0.659 *		0.857 *				
CSI Score				0.504 **											
McGill Score			0.504 **		0.382 *	0.687 **	0.659 *								
Pain Duration (months)				−0.382 *											
**Multitasking**															
Alpha		0.758 ***					0.802 **					0.786 *			
Beta	0.758 ***					0.802 **			0.582 *		0.786 *				
CSI Score				0.504 **											
McGill Score			0.504 **		0.382 *		0.582 *								
Pain Duration (months)				0.382 *											
**Resting-State**															
Alpha		0.563 **					0.824 ***					0.929 **			
Beta	0.563 **					0.824 ***					0.929 **				
CSI Score				0.504 **											
McGill Score			0.504 **		0.382 *										
Pain Duration (months)				0.382 *											

Asterisks specify the level of significance: */**/***—*p* < 0.05/0.01/0.001.

## Data Availability

The data presented in this study are available at the request of the corresponding authors for privacy and ethical reasons.

## References

[1] Mills S.E.E., Nicolson K.P., Smith B.H. (2019). Chronic pain: A review of its epidemiology and associated factors in population-based studies. Br. J. Anaesth..

[2] Raja S.N., Carr D.B., Cohen M., Finnerup N.B., Flor H., Gibson S., Keefe F.J., Mogil J.S., Ringkamp M., Sluka K.A. (2020). The Revised International Association for the Study of Pain Definition of pain: Concepts, challenges, and compromises. Pain.

[3] Reckziegel D., Vachon-Presseau E., Petre B., Schnitzer T.J., Baliki M.N., Apkarian A.V. (2019). Deconstructing biomarkers for chronic pain: Context and hypothesis dependent biomarker types in relation to chronic pain. Pain.

[4] GBD 2016 Disease and Injury Incidence and Prevalence Collaborators (2017). Global, regional, and national incidence, prevalence, and years lived with disability for 328 diseases and injuries for 195 countries, 1990–2016: A systematic analysis for the Global Burden of Disease Study 2016. Lancet.

[5] Kuner R., Flor H. (2016). Structural plasticity and reorganisation in chronic pain. Nat. Rev. Neurosci..

[6] Volcheck M.M., Graham S.M., Fleming K.C., Mohabbat A.B., Luedtke C.A. (2023). Central sensitization, chronic pain, and other symptoms: Better understanding, better management. Clevel. Clin. J. Med..

[7] Baliki M.N., Apkarian A.V. (2015). Nociception, Pain, Negative Moods, and Behavior Selection. Neuron.

[8] Farmer M.A., Baliki M.N., Apkarian A.V. (2012). A dynamic network perspective of chronic pain. Neurosci. Lett..

[9] Yoo Y.M., Kim K.H. (2024). Current understanding of nociplastic pain. Korean J. Pain.

[10] Danno D., Imai N., Kitamura S., Ishizaki K., Kikui S., Takeshima T. (2024). Efficacy of galcanezumab in migraine central sensitization. Sci. Rep..

[11] Lepri B., Romani D., Storari L., Barbari V. (2023). Effectiveness of Pain Neuroscience Education in Patients with Chronic Musculoskeletal Pain and Central Sensitization: A Systematic Review. Int. J. Environ. Res. Public Health.

[12] Rueda M.R., Moyano S., Rico-Picó J. (2023). Attention: The grounds of self-regulated cognition. Wiley Interdiscip. Rev. Cogn. Sci..

[13] Hauck M., Lorenz J., Engel A.K. (2007). Attention to painful stimulation enhances γ-band activity and synchronization in human sensorimotor cortex. J. Neurosci..

[14] Bushnell M.C., Ceko M., Low L.A. (2013). Cognitive and emotional control of pain and its disruption in chronic pain. Nat. Rev. Neurosci..

[15] Eccleston C., Crombez G. (1999). Pain demands attention: A cognitive-affective model of the interruptive function of pain. Psychol. Bull..

[16] Oosterman J.M., Derksen L.C., van Wijck A.J.M., Veldhuijzen D.S., Kessels R.P.C. (2011). Memory Functions in Chronic Pain. Clin. J. Pain.

[17] Higgins D.M., Martin A.M., Baker D.G., Vasterling J.J., Risbrough V. (2017). The Relationship between Chronic Pain and Neurocognitive Function. Clin. J. Pain.

[18] Berryman C., Stanton T.R., Jane Bowering K., Tabor A., McFarlane A., Lorimer Moseley G. (2013). Evidence for working memory deficits in chronic pain: A systematic review and meta-analysis. Pain.

[19] Berryman C., Stanton T.R., Bowering K.J., Tabor A., McFarlane A., Moseley G.L. (2014). Do people with chronic pain have impaired executive function? A meta-analytical review. Clin. Psychol. Rev..

[20] Sobott N., Crowther M.E., Vincent G.E., Belavý D.L., Buntine P., Ferguson S.A., Mundell N.L., Sprajcer M., Tagliaferri S.D., Tait J.L. (2025). Chronic low back pain is associated with compromised cognitive function: A systematic review and meta-analysis. J. Pain.

[21] Mussigmann T., Bardel B., Lefaucheur J.P. (2022). Resting-state electroencephalography (EEG) biomarkers of chronic neuropathic pain. A systematic review. Neuroimage.

[22] Zebhauser P.T., Hohn V.D., Ploner M. (2023). Resting-state electroencephalography and magnetoencephalography as biomarkers of chronic pain: A systematic review. Pain.

[23] Nawaz R., Suen H., Ullah R., Purcell M., Diggin S., McCaughey E., Vuckovic A. (2024). Electroencephalography Longitudinal Markers of Central Neuropathic Pain Intensity in Spinal Cord Injury: A Home-Based Pilot Study. Biomedicines.

[24] Imai H., Kanie Y., Yoshimoto S., Yamamoto N., Furuya M., Fujimori T., Okada S. (2025). Classification accuracy of pain intensity induced by leg blood flow restriction during walking using machine learning based on electroencephalography. Sci. Rep..

[25] Shirvalkar P., Prosky J., Chin G., Ahmadipour P., Sani O.G., Desai M., Schmitgen A., Dawes H., Shanechi M.M., Starr P.A. (2023). First-in-human prediction of chronic pain state using intracranial neural biomarkers. Nat. Neurosci..

[26] You L., Yang B., Lu X., Yang A., Zhang Y., Bi X., Zhou S. (2025). Similarities and differences between chronic primary pain and depression in brain activities: Evidence from resting-state microstates and auditory Oddball task. Behav. Brain Res..

[27] Camargo L., Pacheco-Barrios K., Marques L.M., Caumo W., Fregni F. (2024). Adaptive and Compensatory Neural Signatures in Fibromyalgia: An Analysis of Resting-State and Stimulus-Evoked EEG Oscillations. Biomedicines.

[28] Neblett R., Cohen H., Choi Y., Hartzell M.M., Williams M., Mayer T.G., Gatchel R.J. (2013). The Central Sensitization Inventory (CSI): Establishing Clinically Significant Values for Identifying Central Sensitivity Syndromes in an Outpatient Chronic Pain Sample. J. Pain.

[29] Melzack R. (1975). The McGill Pain Questionnaire: Major properties and scoring methods. Pain.

[30] Robbins T.W., James M., Owen A.M., Sahakian B.J., Lawrence A.D., McInnes L., Rabbitt P.M. (1998). A study of performance on tests from the CANTAB battery sensitive to frontal lobe dysfunction in a large sample of normal volunteers: Implications for theories of executive functioning and cognitive aging. Cambridge Neuropsychological Test Automated Battery. J. Int. Neuropsychol. Soc..

[31] Robbins T.W., James M., Owen A.M., Sahakian B.J., McInnes L., Rabbitt P. (1994). Cambridge Neuropsychological Test Automated Battery (CANTAB): A factor analytic study of a large sample of normal elderly volunteers. Dementia.

[32] Dinh S.T., Nickel M.M., Tiemann L., May E.S., Heitmann H., Hohn V.D., Edenharter G., Utpadel-Fischler D., Tölle T.R., Sauseng P. (2019). Brain dysfunction in chronic pain patients assessed by resting-state electroencephalography. Pain.

[33] Tadel F., Baillet S., Mosher J.C., Pantazis D., Leahy R.M. (2011). Brainstorm: A User-Friendly Application for MEG/EEG Analysis. Comput. Intell. Neurosci..

[34] Welch P. (1967). The use of fast Fourier transform for the estimation of power spectra: A method based on time averaging over short, modified periodograms. IEEE Trans. Audio Electroacoust..

[35] JASP Team (2022). JASP.

[36] Pfurtscheller G. (1977). Graphical display and statistical evaluation of event-related desynchronization (ERD). Electroencephalogr. Clin. Neurophysiol..

[37] Michels L., Bucher K., Lüchinger R., Klaver P., Martin E., Jeanmonod D., Brandeis D. (2010). Simultaneous EEG-fMRI during a working memory task: Modulations in low and high frequency bands. PLoS ONE.

[38] Chafee M.V., Goldman-Rakic P.S. (1998). Matching patterns of activity in primate prefrontal area 8a and parietal area 7ip neurons during a spatial working memory task. J. Neurophysiol..

[39] Rottschy C., Caspers S., Roski C., Reetz K., Dogan I., Schulz J.B., Zilles K., Laird A.R., Fox P.T., Eickhoff S.B. (2013). Differentiated parietal connectivity of frontal regions for “what” and ”where” memory. Brain Struct. Funct..

[40] Ruspantini I., Mäki H., Korhonen R., D’Ausilio A., Ilmoniemi R.J. (2011). The functional role of the ventral premotor cortex in a visually paced finger tapping task: A TMS study. Behav. Brain Res..

[41] Proskovec A.L., Wiesman A.I., Heinrichs-Graham E., Wilson T.W. (2018). Beta Oscillatory Dynamics in the Prefrontal and Superior Temporal Cortices Predict Spatial Working Memory Performance. Sci. Rep..

[42] Sochůrková D., Rektor I., Jurák P., Stancák A. (2006). Intracerebral recording of cortical activity related to self-paced voluntary movements: A Bereitschaftspotential and event-related desynchronization/synchronization. SEEG study. Exp. Brain Res..

[43] Boiten F., Sergeant J., Geuze R. (1992). Event-related desynchronization: The effects of energetic and computational demands. Electroencephalogr. Clin. Neurophysiol..

[44] Dujardin K., Bourriez J.L., Guieu J.D. (1995). Event-related desynchronization (ERD) patterns during memory processes: Effects of aging and task difficulty. Electroencephalogr. Clin. Neurophysiol..

[45] Klimesch W., Doppelmayr M., Russegger H., Pachinger T., Schwaiger J. (1998). Induced alpha band power changes in the human EEG and attention. Neurosci. Lett..

[46] Klimesch W., Sauseng P., Hanslmayr S. (2007). EEG alpha oscillations: The inhibition-timing hypothesis. Brain Res. Rev..

[47] Chen Y.T., van Ede F., Kuo B.C. (2022). Alpha Oscillations Track Content-Specific Working Memory Capacity. J. Neurosci..

[48] Kim S.J., Yang K., Kim D. (2023). Quantitative electroencephalography as a potential biomarker in migraine. Brain Behav..

[49] Bridge H., Stagg C.J., Near J., Lau C.I., Zisner A., Cader M.Z. (2015). Altered neurochemical coupling in the occipital cortex in migraine with visual aura. Cephalalgia.

[50] Wu X., Han S., Yang Y., Dai H., Wu P., Zhao H., Jin X., Li Y. (2022). Decreased Brain GABA Levels in Patients with Migraine Without Aura: An Exploratory Proton Magnetic Resonance Spectroscopy Study. Neuroscience.

[51] Schreckenberger M., Lange-Asschenfeldt C., Lochmann M., Mann K., Siessmeier T., Buchholz H.G., Bartenstein P., Gründer G. (2004). The thalamus as the generator and modulator of EEG alpha rhythm: A combined PET/EEG study with lorazepam challenge in humans. Neuroimage.

[52] Huang J., Wilkins A. (2021). The Functional Network of the Visual Cortex Is Altered in Migraine. Vision.

[53] de Tommaso M., Trotta G., Vecchio E., Ricci K., Siugzdaite R., Stramaglia S. (2017). Brain networking analysis in migraine with and without aura. J. Headache Pain.

[54] Lee M.J., Park B.Y., Cho S., Kim S.T., Park H., Chung C.S. (2019). Increased connectivity of pain matrix in chronic migraine: A resting-state functional MRI study. J. Headache Pain.

[55] Gou C., Yang S., Hou Q., Rudder P., Tanglay O., Young I., Peng T., He W., Yang L., Osipowicz K. (2023). Functional connectivity of the language area in migraine: A preliminary classification model. BMC Neurol..

[56] London R.E., Benwell C.S.Y., Cecere R., Quak M., Thut G., Talsma D. (2022). EEG alpha power predicts the temporal sensitivity of multisensory perception. Eur. J. Neurosci..

[57] Pinheiro E.S., de Queirós F.C., Montoya P., Santos C.L., do Nascimento M.A., Ito C.H., Silva M., Nunes Santos D.B., Benevides S., Miranda J.G. (2016). Electroencephalographic Patterns in Chronic Pain: A Systematic Review of the Literature. PLoS ONE.

[58] De Pascalis V., Scacchia P., Papi B., Corr P.J. (2019). Changes of EEG band oscillations to tonic cold pain and the behavioral inhibition and fight-flight-freeze systems. Personal. Neurosci..

[59] Harmony T. (2013). The functional significance of delta oscillations in cognitive processing. Front. Integr. Neurosci..

[60] Knyazev G.G. (2007). Motivation, emotion, and their inhibitory control mirrored in brain oscillations. Neurosci. Biobehav. Rev..

[61] de Vries I.E.J., Savran E., van Driel J., Olivers C.N.L. (2019). Oscillatory Mechanisms of Preparing for Visual Distraction. J. Cogn. Neurosci..

[62] Riddle J., Scimeca J.M., Cellier D., Dhanani S., D’Esposito M. (2020). Causal Evidence for a Role of Theta and Alpha Oscillations in the Control of Working Memory. Curr. Biol..

[63] Hoegh M., Seminowicz D.A., Graven-Nielsen T. (2019). Delayed effects of attention on pain sensitivity and conditioned pain modulation. Eur. J. Pain.

[64] Kucyi A., Salomons T.V., Davis K.D. (2013). Mind wandering away from pain dynamically engages antinociceptive and default mode brain networks. Proc. Natl. Acad. Sci. USA.

[65] Mac Goris J.L., Todd J., Clarke P.J.F., Hughes A.M., Vögele C., Van Ryckeghem D.M.L. (2024). The role of attention bias malleability in experiencing pain and associated disability. PeerJ.

[66] Magosso E., Borra D. (2024). The strength of anticipated distractors shapes EEG alpha and theta oscillations in a Working Memory task. Neuroimage.

[67] Engel A.K., Fries P. (2010). Beta-band oscillations—Signalling the status quo?. Curr. Opin Neurobiol..

[68] Wessel J.R., Aron A.R. (2017). On the Globality of Motor Suppression: Unexpected Events and Their Influence on Behavior and Cognition. Neuron.

[69] Di Dona G., Ronconi L. (2023). Beta oscillations in vision: A (preconscious) neural mechanism for the dorsal visual stream?. Front. Psychol..

[70] May E.S., Nickel M.M., Ta Dinh S., Tiemann L., Heitmann H., Voth I., Tölle T.R., Gross J., Ploner M. (2018). Prefrontal gamma oscillations reflect ongoing pain intensity in chronic back pain patients. Hum. Brain Mapp..

[71] Jensen O., Kaiser J., Lachaux J.P. (2007). Human gamma-frequency oscillations associated with attention and memory. Trends Neurosci..

[72] Popov T., Jensen O., Schoffelen J.M. (2018). Dorsal and ventral cortices are coupled by cross-frequency interactions during working memory. Neuroimage.

[73] Brzezicka A., Kamiński J., Reed C.M., Chung J.M., Mamelak A.N., Rutishauser U. (2019). Working Memory Load-related Theta Power Decreases in Dorsolateral Prefrontal Cortex Predict Individual Differences in Performance. J. Cogn. Neurosci..

[74] de Vries I.E.J., van Driel J., Karacaoglu M., Olivers C.N.L. (2018). Priority Switches in Visual Working Memory are Supported by Frontal Delta and Posterior Alpha Interactions. Cereb Cortex.

[75] Zelmann R., Lina J.M., Schulze-Bonhage A., Gotman J., Jacobs J. (2014). Scalp EEG is not a blur: It can see high frequency oscillations although their generators are small. Brain Topogr..

[76] Muthukumaraswamy S.D. (2013). High-frequency brain activity and muscle artifacts in MEG/EEG: A review and recommendations. Front. Hum. Neurosci..

[77] Whitham E.M., Lewis T., Pope K.J., Fitzgibbon S.P., Clark C.R., Loveless S., DeLosAngeles D., Wallace A.K., Broberg M., Willoughby J.O. (2008). Thinking activates EMG in scalp electrical recordings. Clin. Neurophysiol..

[78] Gaižauskaitė R., Gladutytė L., Zelionkaitė I., Čėsnaitė E., Busch N.A., Grikšienė R. (2024). The search for the relationship between female hormonal status, alpha oscillations, and aperiodic features of resting state EEG. Int. J. Psychophysiol..

[79] Gaižauskaitė R., Gladutytė L., Zelionkaitė I., Grikšienė R. (2025). Exploring the role of sex, sex steroids, menstrual cycle, and hormonal contraception use in visual working memory: Insights from behavioral and EEG analyses. Int. J. Psychophysiol..

[80] Ramos-Loyo J., González-Garrido A.A., Llamas-Alonso L.A., Sequeira H. (2022). Sex differences in cognitive processing: An integrative review of electrophysiological findings. Biol. Psychol..

[81] Sorge R.E., Totsch S.K. (2016). Sex Differences in Pain. J. Neurosci. Res..

[82] Birkinshaw H., Friedrich C.M., Cole P., Eccleston C., Serfaty M., Stewart G., White S., Moore R.A., Phillippo D., Pincus T. (2023). Antidepressants for pain management in adults with chronic pain: A network meta-analysis. Cochrane Libr..

[83] Narayan S.W., Naganathan V., Vizza L., Underwood M., Ivers R., McLachlan A.J., Zhou L., Singh R., Tao S., Xi X. (2024). Efficacy and Safety of Antidepressants for Pain in Older Adults: A Systematic Review and Meta-analysis. Br. J. Clin. Pharmacol..

[84] Ferreira G.E., Abdel-Shaheed C., Underwood M., Finnerup N.B., Day R.O., McLachlan A., Eldabe S., Zadro J.R., Maher C.G. (2023). Efficacy, safety, and tolerability of antidepressants for pain in adults: Overview of systematic reviews. BMJ.

[85] Zebhauser P.T., Bott F., Ávila C.G., Heitmann H., May E.S., Tiemann L., Baki E., Tölle T.R., Ploner M. (2025). Effects of centrally acting analgesics on resting-state electroencephalography biomarker candidates of chronic pain. J. Pain.

